# Retracted: Analysis Model of the Impact of Refined Intervention in Operating Room on Patients' Recovery Quality and Complications after Thoracic Surgery Based on Deep Neural Network

**DOI:** 10.1155/2022/9753430

**Published:** 2022-11-24

**Authors:** Journal of Healthcare Engineering

*Journal of Healthcare Engineering* has retracted the article titled “Analysis Model of the Impact of Refined Intervention in Operating Room on Patients' Recovery Quality and Complications after Thoracic Surgery Based on Deep Neural Network” [1] due to concerns that the peer review process has been compromised.

Following an investigation conducted by the Hindawi Research Integrity team [2], significant concerns were identified with the peer reviewers assigned to this article; the investigation has concluded that the peer review process was compromised. We therefore can no longer trust the peer review process, and the article is being retracted with the agreement of the Chief Editor.

## References

[1] He J., Wen J. (2021). Analysis Model of the Impact of Refined Intervention in Operating Room on Patients’ Recovery Quality and Complications after Thoracic Surgery Based on Deep Neural Network. *Journal of Healthcare Engineering*.

[2] Ferguson L. (2022). Advancing Research Integrity Collaboratively and with Vigour. https://www.hindawi.com/post/advancing-research-integrity-collaboratively-and-vigour/.

